# A Combined Serum Biomarker Panel for Early Prediction of Response to Anti-TNF Therapy in Rheumatoid Arthritis: Toward a Precision Medicine Approach

**DOI:** 10.3390/diagnostics16111627

**Published:** 2026-05-26

**Authors:** Bogdan Ion Gavrila, Claudia Ciofu, Marilena Stoian

**Affiliations:** Carol Davila University of Medicine and Pharmacy, Department of Internal Medicine and Rheumatology, Dr. I. Cantacuzino Clinical Hospital, 050474 Bucharest, Romaniamarinela.stoian@umfcd.ro (M.S.)

**Keywords:** rheumatoid arthritis, biomarkers, predictors, treatment response, anti-TNFα

## Abstract

**Background/Objectives:** Response to TNF inhibitors in RA remains heterogeneous and reliable predictors of treatment response are still lacking. Biomarker-based stratification may improve therapeutic decision-making and aligns with the emerging paradigm of precision medicine. **Methods:** We conducted a prospective observational study including 64 biologic-naïve patients with active RA being inadequately controlled by csDMARDs. All patients initiated anti-TNF therapy and were followed for 12 months. Clinical response was assessed at 6 and 12 months using EULAR response criteria based on DAS28-CRP. Baseline serum levels of classical biomarkers (RF type IgM, RF type IgA, anti-CCP) and additional biomarkers (anti-MCV,14-3-3η protein, COMP) were evaluated. Logistic regression analyses were performed to identify predictors of treatment response. **Results:** At 6 months, 7 patients were classified as non-responders, 38 as moderate responders, and 19 as good responders Lower baseline levels of RF isotypes, anti-CCP antibodies, 14-3-3η protein, and COMP were associated with favorable clinical response at 6 months. Baseline anti-CCP and 14-3-3η protein levels emerged as significant predictors in univariate analysis. Multivariate logistic regression yielded a predictive model incorporating anti-CCP, 14-3-3η protein, and COMP, achieving an overall prediction accuracy of 89.1%. At 12 months, baseline RF isotypes remained associated with treatment response, whereas the predictive value of other biomarkers diminished. Longitudinal analysis demonstrated significant reductions mainly for classical autoantibody levels under anti-TNF α inhibitors. **Conclusions:** A combined serum biomarker panel may support early prediction of response to anti-TNF therapy in RA. These findings highlight the potential of integrated biomarker-based stratification to optimize therapeutic decisions and support the implementation of precision medicine approaches in RA.

## 1. Introduction

Rheumatoid arthritis (RA) is a chronic, systemic autoimmune inflammatory disease characterized by persistent synovitis, progressive joint destruction, functional disability, and increased cardiovascular morbidity and mortality [1,2]. Affecting approximately 0.5–1% of the adult population worldwide, RA represents one of the most prevalent chronic inflammatory rheumatic diseases. It develops through a complex interaction between genetic predisposition, environmental exposures, and alterations in immune regulation. One of the key mechanisms implicated in this process is the loss of tolerance toward proteins that undergo post-translational modifications, particularly citrullination. This biochemical modification is catalyzed by peptidylarginine deiminase enzymes, which convert arginine residues into citrulline, leading to conformational changes in protein structure. As a result, these modified proteins may be recognized as non-self by the immune system, promoting the generation of anti-citrullinated protein antibodies (ACPAs). These autoantibodies contribute to disease pathogenesis by forming immune complexes that activate complement pathways and sustain synovial inflammation. Moreover, citrullinated antigens can enhance the activation of key effector cells, including macrophages and fibroblast-like synoviocytes, thereby amplifying inflammatory responses and promoting joint damage. Altogether, these processes highlight the central role of citrullination in the initiation and progression of rheumatoid arthritis and provide a biological rationale for the clinical relevance of ACPA-related biomarkers [3].

Beyond its clinical manifestations, RA imposes a substantial socioeconomic burden, driven by long-term pharmacological treatment, healthcare utilization, work disability, and productivity loss, particularly in patients with active or treatment-refractory disease [4,5].

The introduction of biologic disease-modifying antirheumatic drugs (bDMARDs), especially tumor necrosis factor-alpha (TNFα) inhibitors, has markedly improved disease control, structural outcomes, and quality of life in patients with moderate-to-severe RA who have inadequately responded to conventional synthetic disease-modifying antirheumatic drugs (csDMARDs) [6,7].

Over the past two decades, the therapeutic landscape of RA has expanded substantially with the development of multiple biologic and targeted synthetic agents directed against distinct immunological pathways. These include inhibitors of tumor necrosis factor-alpha (TNFα), interleukin-6 (IL-6) receptor antagonists, costimulation blockers such as CTLA-4–Ig, B-cell-depleting therapies, and, more recently, Janus kinase (JAK) inhibitors. Among these, TNFα inhibitors were the first biologic agents introduced into clinical practice and remain the most widely used class due to their well-established efficacy and long-term safety profile. By neutralizing TNFα, a key cytokine involved in synovial inflammation and joint destruction, these agents effectively reduce disease activity and prevent structural damage.

However, a significant proportion of patients experience primary non-response or secondary loss of efficacy, underscoring the marked inter-individual variability in therapeutic outcomes. Identifying predictive biomarkers remains a critical unmet need, with potential implications for personalized treatment selection, improved clinical outcomes and cost-effective use of biologic agents [8,9].

Classical serological markers, including rheumatoid factor (RF) and anti-cyclic citrullinated peptide (anti-CCP) antibodies, have been extensively investigated not only for diagnostic and prognostic purposes but also for their potential predictive value regarding response to TNFα inhibitors, yielding heterogeneous results across studies [10,11]. Additional citrullination-related antibodies, such as anti-mutated citrullinated vimentin (anti-MCV), have been proposed as complementary markers reflecting distinct immunopathogenic pathways, although their predictive role remains controversial [12].

More recently, novel biomarkers associated with inflammation, joint damage, and intracellular signaling pathways have gained attention. Among these, 14-3-3 eta protein has been implicated in the amplification of pro-inflammatory signaling cascades [13], while cartilage oligomeric matrix protein (COMP) reflects cartilage turnover and structural disease activity. Both have been suggested as potential adjunctive biomarkers to improve prediction models for biologic treatment response in RA [14].

The objectives of the study were the following:

1. To evaluate the predictive value of classical diagnostic biomarkers (RF IgM and IgA isotypes, anti-CCP) for treatment response to anti-TNFα biologic therapy.
2. To assess the predictive potential of selected novel biomarkers with possible diagnostic and clinical relevance (anti-MCV, COMP, and 14-3-3 eta protein) in relation to response to anti-TNFα therapy.
3. To identify and develop a pre-treatment prediction model for biologic treatment response.
4. To monitor longitudinal changes in serum biomarker levels during biologic therapy, in order to explore the concept of immunological remission beyond clinical remission

## 2. Materials and Methods

Patients numbered 64 with clinically and biologically active RA that was inadequately controlled by conventional csDMARDs, administered either as monotherapy or in combination. All patients were biologic-naïve at study entry.

Biologic therapy with anti-TNFα inhibitors was initiated as follows: 18 patients received Adalimumab, 16 Etanercept, 13 Certolizumab pegol, and 17 patients were treated with biosimilars of Infliximab.

All participants provided written informed consent prior to study inclusion. The study protocol was approved by the Ethics Committee of Dr. I. Cantacuzino Clinical Hospital, Bucharest, Romania.

### 2.1. Inclusion Criteria

Patients were eligible for inclusion if they met all of the following criteria.

- Fulfillment of the eligibility criteria established by the National Health Insurance House expert committee in Romania for approval of biologic therapy in rheumatoid arthritis, including the following:
  ○ Active disease despite ongoing treatment, defined by the presence of at least five joints with active synovitis (tenderness and swelling) and DAS28 score > 5.1
  ○ The presence of at least two of the following three criteria:
    ▪ Morning stiffness, lasting more than 60 min;
    ▪ Erythrocyte sedimentation rate (ESR) > 28 mm/h;
    ▪ C-reactive protein (CRP) levels exceeding three times the upper limit of normal.
- Previous treatment with at least two csDMARDs for a minimum duration of 12 weeks, one of which was methotrexate (MTX), unless contraindicated.
- Age ≥ 18 years.

### 2.2. Exclusion Criteria

Patients were excluded from the study based on the following criteria:

- Presence of concomitant infections;
- Development of adverse reactions during the course of treatment;
- Patients who discontinued treatment before the 6- or 12-month evaluation were excluded from the final analysis.

### 2.3. Assessment of Treatment Respone

Treatment response was assessed according to the EULAR response criteria at visit 2 (6 months) and visit 3 (12 months). Based on changes in disease activity and achieved DAS28 (CRP) values, patients were classified into three categories: good responders, moderate responders, and non-responders.

### 2.4. Research Infrastructure and Laboratory Assessments

Collected biological samples were processed and analyzed in collaboration with the “Victor Babeș” National Institute of Research and Development in Pathology and Biomedical Sciences, Bucharest, Romania.

C-reactive protein (CRP) levels were measured using a nephelometric method, with normal values defined as <3.8 mg/L. Serum levels of all six biomarkers were determined using enzyme-linked immunosorbent assay (ELISA), including RF IgM and IgA isotypes, anti-CCP, anti-MCV, 14-3-3 eta protein, and COMP.

The reference ranges were as follows: RF IgM and IgA < 13 U/mL, anti-CCP < 40 ng/mL, anti-MCV < 9 ng/mL, 14-3-3 eta protein < 0.3 ng/mL, and COMP 0–700 ng/mL.

Statistical analyses were performed using SPSS software 25 (IBM Corp., Armonk, NY, USA), according to standard statistical procedures.

Data were expressed as percentages and means ± standard deviations.

Where appropriate, correction for multiple comparisons was performed using Bonferroni-adjusted analyses, particularly following Kruskal–Wallis testing and subgroup comparisons. A value of *p* ≤ 0.05 was considered statistically significant.

## 3. Results

The mean age of the study population was 57.55 ± 9.43 years; female patients predominated, accounting for 92.2%.

Among other diseases despite RA, high blood pressure was the most frequently observed condition (57.8%). Metabolic comorbidities, including diabetes (20.3%) and dyslipidemia (32.8%), were also identified in a substantial proportion of patients (Table 1).

Regarding csDMARDs, the most used was MTX-35 (54.7%) of the 64 subjects who were receiving treatment with this csDMARD at the time of initiation of biological treatment. The next csDMARD used as first-line therapy after MTX was Leflunomide (LEF); 25 (39.1%) patients were receiving this treatment.

Regarding second-line csDMARDs, Sulfasalazine (SSZ) was the most frequently used (54.6%), followed by LEF (39%). Before starting biological therapy, 21 patients were receiving treatment with glucocorticoids combined with csDMARDs (Table 2).

At visit 2 (6 months), among patients treated with anti-TNFα agents, 7 patients were classified as non-responders, 38 achieved a moderate response, and 19 achieved a good response. One patient was excluded from the analysis at this visit due to the occurrence of side events.

Smoking was also evaluated. Within the group, after comparing the percentages of smokers/former/non-smokers and the response at 6 months, we recorded differences close to significant (*p* = 0.056). Thus, we observed that in the group of patients who obtained a good response at 6 months, 89.5% of them were non-smokers, only 28.6% of the non-responders declaring themselves non-smokers. We also observed a high percentage of smokers in the non-responders group (71.4%).

When dividing patients only into non-responders and responders, at visit 2 we observed significant differences (*p* = 0.017) between the percentages of smokers/former/non-smokers and the response at 6 months. It was observed that the response was dominated by non-smokers, and non-responders by smokers (Table 3). For visit 3 we did not record significant differences.

### 3.1. Baseline Biomarkers Titers and 6-Month Response

Analyzing baseline serum titers of biomarkers in relation to treatment response at 6 months among patients treated with anti-TNFα agents, several significant differences were identified.

Baseline RF IgM levels differed significantly across response categories (Kruskal–Wallis test with Bonferroni correction, *p* = 0.0162). Patients who achieved a good response at visit 2 had lower mean baseline RF IgM levels (51.36 ± 95.36 U/mL) compared with moderate responders (157.22 ± 131.48 U/mL) and non-responders (132.35 ± 99.60 U/mL).

Similarly, baseline RF IgA levels were significantly lower in good responders (22.45 ± 61.26 U/mL) compared with non-responders (122.81 ± 99.88 U/mL) and moderate responders (102.08 ± 128.33 U/mL) (Kruskal–Wallis test, *p* = 0.0334).

Baseline anti-CCP antibody levels also differed significantly between response groups (Kruskal–Wallis test with Bonferroni correction, *p* = 0.0001). Lower baseline anti-CCP levels were observed in good responders (60.82 ± 26.33 ng/mL) compared with moderate responders (113.65 ± 50.45 ng/mL) and non-responders (146.16 ± 41.69 ng/mL).

Baseline anti-MCV levels were lower in good responders (33.77 ± 113.07 ng/mL) than in non-responders (74.04 ± 47.95 ng/mL) and moderate responders (80.06 ± 149.54 ng/mL); however, these differences did not reach statistical significance (Kruskal–Wallis test with Bonferroni correction, *p* = 0.4591).

Regarding 14-3-3 eta protein, significant differences in baseline levels were observed among response groups (Kruskal–Wallis test with Bonferroni correction, *p* = 0.0451), with lower values in moderate responders (0.28 ± 0.47 ng/mL) and good responders (0.51 ± 0.58 ng/mL) compared with non-responders (0.99 ± 0.89 ng/mL).

**Figure 1:**
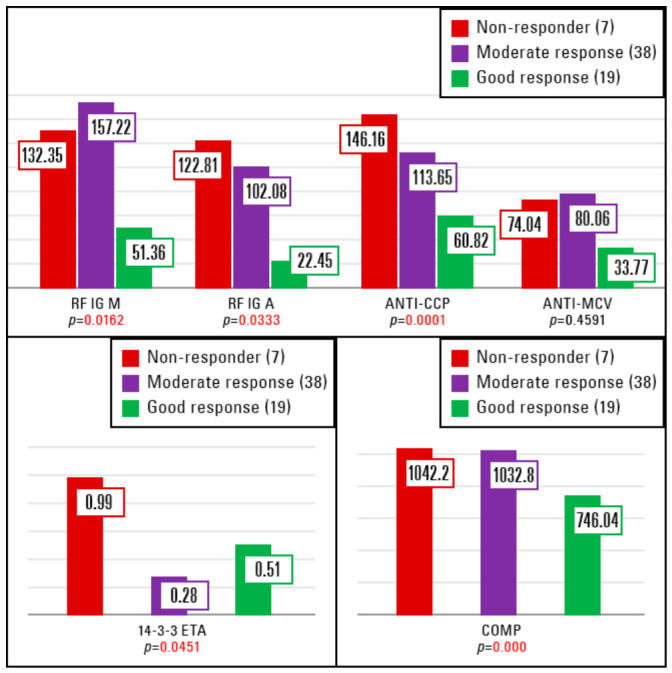
Baseline titers and treatment EULAR response at 6 months.

Baseline COMP levels also differed significantly between response categories (Kruskal–Wallis test, *p* < 0.001). Patients with a good response had lower baseline COMP levels (746.04 ± 130.10 ng/mL) compared with moderate responders (1032.80 ± 188.67 ng/mL) and non-responders (1042.20 ± 181.72 ng/mL) (Figure 1).

### 3.2. Baseline Biomarkers Titers and 12-Month Response

At visit 3 (12 months), among the 56 patients who remained in the study, 1 patient was classified as a non-responder, 11 achieved a moderate response, and 44 achieved a good response. Significant differences in treatment response were identified in relation to baseline values of both RF isotypes.

Baseline RF IgM levels differed significantly between response groups, with lower mean values observed in patients with a good response (92.93 ± 120.22 U/mL) compared with moderate responders (231.83 ± 104.83 U/mL) and the non-responder (300.00 ± 0.00 U/mL) (Kruskal–Wallis test with Bonferroni correction, *p* = 0.0103).

Similarly, baseline RF IgA levels were significantly lower in good responders (49.96 ± 98.08 U/mL) compared with moderate responders (162.80 ± 133.10 U/mL) (*p* = 0.0024).

Lower baseline mean values were also observed for anti-mutated citrullinated vimentin (anti-MCV) antibodies and anti-cyclic citrullinated peptide (anti-CCP) antibodies in patients with a good response compared with the other response groups; however, these differences were not statistically significant.

Baseline levels of 14-3-3 eta protein were surprisingly higher in patients who achieved a good response at visit 3, but no statistically significant differences were identified between response categories.

**Figure 2:**
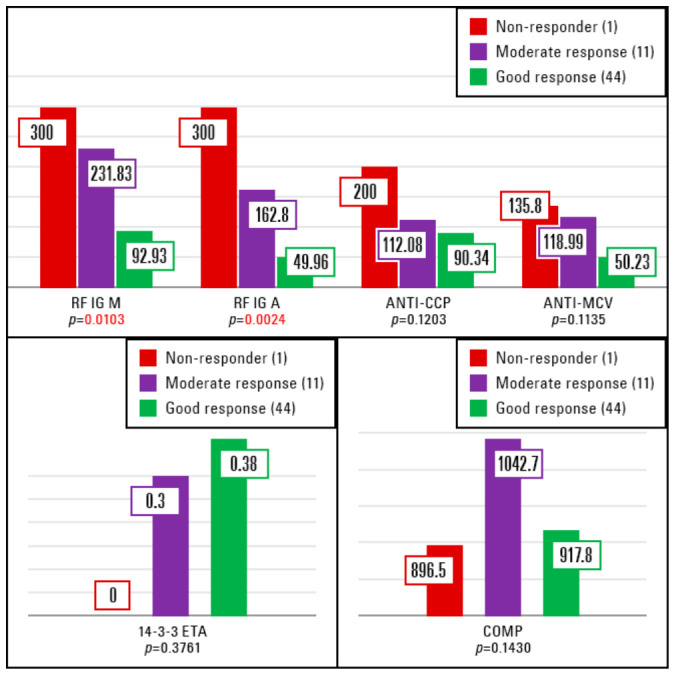
Baseline titers and treatment EULAR response at 12 months.

Regarding COMP, patients with a good response at this evaluation exhibited lower baseline levels compared with those with a moderate response; however, these differences did not reach statistical significance (Figure 2).

When patients were divided into two groups according to treatment response at visit 2 (non-responders, *n* = 7; responders, *n* = 57), lower mean baseline values were observed for all six immunological biomarkers in the responder group compared with non-responders.

**Figure 3:**
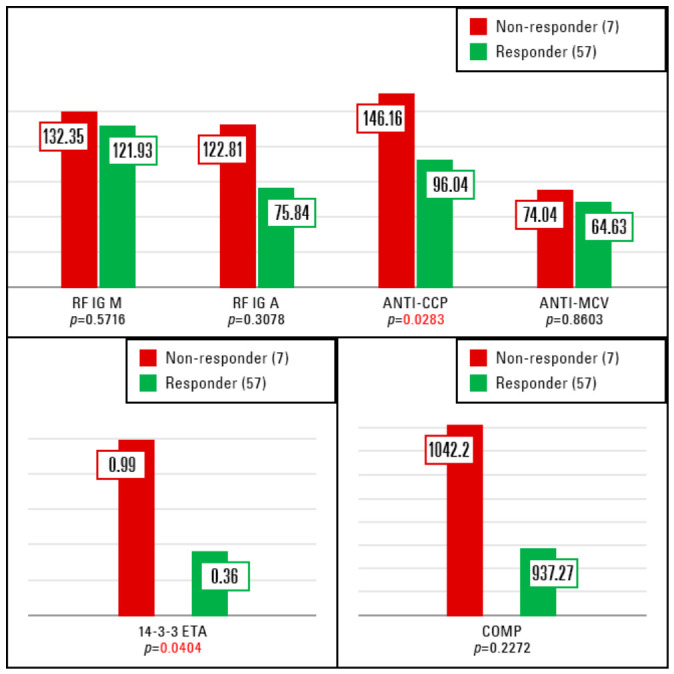
Baseline titers and 6 months treatment response/non-responders.

Statistically significant differences were identified for baseline 14-3-3 eta protein levels, which were lower in responders (0.36 ± 0.52 ng/mL) compared with non-responders (0.99 ± 0.89 ng/mL; *p* = 0.0404). Significant differences were also observed for baseline anti-CCP antibody levels, with lower values in responders (96.04 ± 50.36 ng/mL) compared with non-responders (146.16 ± 41.69 ng/mL; *p* = 0.0283) (Figure 3).

When baseline biomarker levels were compared according to treatment response at 12 months (responders, *n* = 55; non-responder, *n* = 1), no statistically significant differences were observed. However, the single non-responder exhibited higher baseline levels for five of the six evaluated biomarkers compared with the responder group.

Identification of predictors for treatment response at 6 months.

Given that, at 6 months, the only parameters demonstrating a predictive value for treatment response—after dichotomizing patients into non-responders and responders—were baseline anti-CCP levels and 14-3-3 eta protein levels; logistic regression analyses were performed to further explore these associations. Owing to the unequal distribution of patients across the three EULAR response categories (non-response, moderate response, and good response), regression analyses were conducted dividing patients into responders and non-responders.

Univariate logistic regression analysis was applied to evaluate potential predictors of treatment response at 6 months in patients treated with anti-TNFα agents. Each variable was initially assessed individually. Baseline anti-CCP antibody levels (*p* = 0.0255, 95% CI 0.9632–0.9976, OR = 0.9802) and baseline 14-3-3 eta protein levels (*p* = 0.0169, 95% CI 0.0722–0.7715, OR = 0.2360) were identified as significant predictors of treatment response.

**Figure 4:**
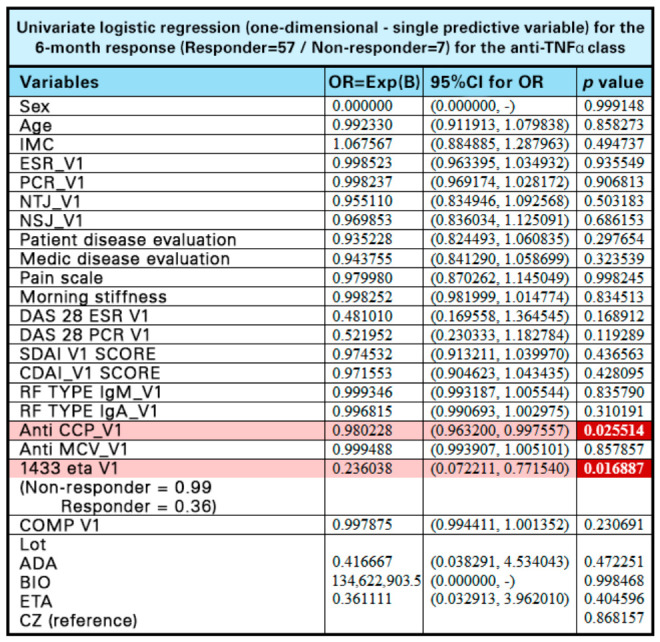
Predictors for 6 month treatment response.

Higher baseline values of either anti-CCP antibodies or 14-3-3 eta protein were associated with a reduced probability of achieving a clinical response at 6 months (Figure 4).

### 3.3. Statistical Model for Predicting 6 Month Response

**Figure 5:**
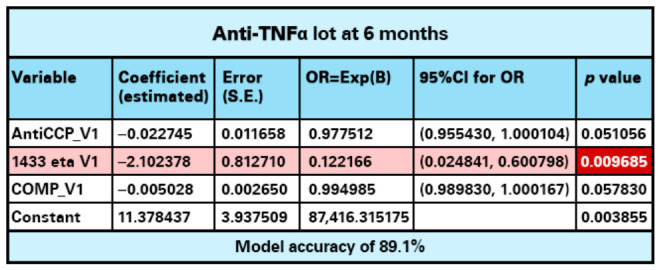
Model for predicting 6 month treatment response.

All previously identified parameters were included in a multivariate logistic regression analysis with multiple predictor variables. A Forward Stepwise (Wald) logistic regression model was applied. The resulting model demonstrated good calibration and an adequate fit to the data, as assessed by the Hosmer–Lemeshow goodness-of-fit test (χ^2^ = 5.79, *p* = 0.67 ≥ 0.05) (Figure 5).

Three parameters remained significant in predicting treatment response at 6 months: baseline anti-CCP levels, 14-3-3 eta protein, and COMP levels. The final model correctly classified 89.1% of patients with respect to treatment response.

Among these predictors, 14-3-3 eta protein demonstrated the strongest impact on the probability of achieving a treatment response, producing the largest change in response likelihood (2.103).

The final predictive model, including only statistically significant variables, is presented below.

### 3.4. Evolution of Serum Biomarker Titers Under Anti-TNF α

In patients treated with anti-TNFα agents, a statistically significant reduction in the mean serum levels of both RF isotypes (IgM and IgA), as well as anti-CCP antibodies, was observed at both 6 and 12 months of treatment.

A decrease in mean serum levels after treatment initiation was also noted for MCV antibodies and 14-3-3 eta protein; however, these changes did not reach statistical significance, with the exception of anti-MCV levels, which showed a significant reduction between baseline (65.66 ± 132.08 ng/mL) and the 12-month evaluation (17.15 ± 27.86 ng/mL; *p* = 0.0312).

**Figure 6:**
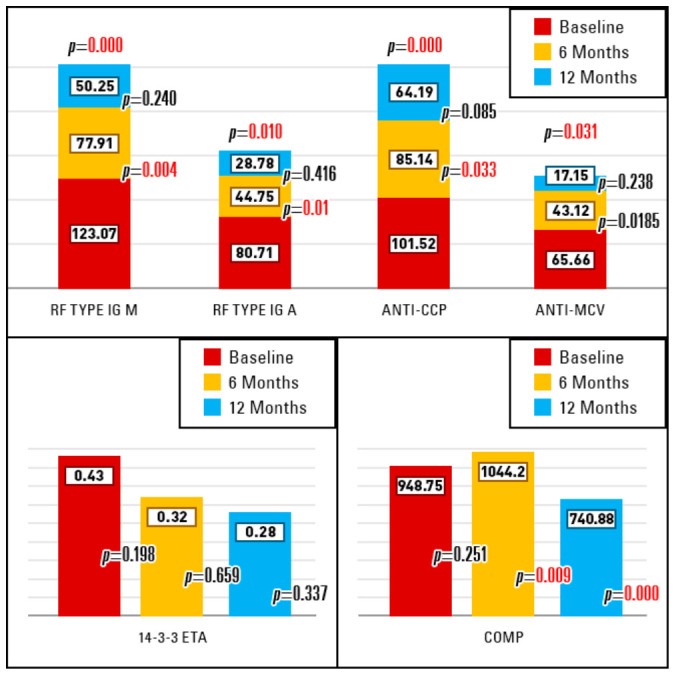
Evolution of biomarkers serum titers under biologic anti-TNFα.

Regarding COMP, mean serum levels increased at 6 months, followed by a subsequent decrease at visit 3. Statistically significant reductions were observed between baseline and 12 months (948.75 ± 215.68 ng/mL vs. 740.88 ± 227.04 ng/mL; *p* < 0.001), as well as between the 6-month and 12-month evaluations (1044.20 ± 674.67 ng/mL vs. 740.88 ± 227.04 ng/mL; *p* = 0.0092) (Figure 6).

## 4. Discussion

The present prospective observational study evaluated the predictive value of both classical and novel immunological biomarkers for treatment response to TNFα inhibitors in patients with active rheumatoid arthritis inadequately controlled by csDMARDs. Our results demonstrate that lower baseline serum levels of classical autoantibodies, particularly RF IgM, RF IgA, and anti-CCP antibodies, as well as selected novel biomarkers such as 14-3-3 eta protein and COMP, were associated with a higher likelihood of achieving a favorable clinical response to anti-TNFα therapy, particularly at the 6-month evaluation. Furthermore, multivariate analysis identified a combined biomarker-based predictive model incorporating anti-CCP antibodies, 14-3-3 eta protein, and COMP levels, supporting the concept that multidimensional biomarker assessment may better capture the biological heterogeneity underlying treatment response in RA than single-marker approaches [15].

In the present study, classical serological biomarkers demonstrated a consistent association with clinical response to anti-TNFα therapy, particularly at the 6-month evaluation. Lower baseline levels of both RF isotypes (IgM and IgA) and anti-CCP antibodies were observed in patients achieving a good or moderate EULAR response, with anti-CCP antibodies emerging as a significant predictor of treatment response in univariate analysis. These findings are in line with previous reports suggesting that a lower autoantibody burden may reflect a less aggressive or immunologically entrenched disease phenotype, which is that more may respond better to cytokine-targeted biologic therapy [16,17].

Although RF and anti-CCP antibodies are primarily established as diagnostic and prognostic markers in rheumatoid arthritis, their potential role in predicting response to biologic therapy has been increasingly explored, with heterogeneous results across studies. Variability in study design, patient populations, disease duration, and definitions of treatment response likely contribute to these inconsistencies [6,7]. Our data support that, while classical autoantibodies alone may not be sufficient as standalone predictors, lower pre-treatment anti-CCP levels—particularly in the context of early treatment evaluation—may identify patients more likely to achieve a favorable clinical response to TNFα inhibition [18].

A key finding of this study is the significant association between baseline serum levels of 14-3-3 eta protein and the clinical response to anti-TNFα therapy at 6 months. Lower pre-treatment levels of this biomarker were observed in responders, and 14-3-3 eta protein emerged as one of the strongest predictors of treatment response in both univariate and multivariate analyses, exerting the greatest impact within the final predictive model [19].

Biologically, 14-3-3 eta protein is involved in the amplification of intracellular inflammatory signaling pathways, including MAPK and NF-κB activation, promoting sustained cytokine production and matrix degradation in rheumatoid arthritis. Elevated baseline levels have been associated with more aggressive disease phenotypes and radiographic progression, suggesting a disease state that may be less dependent on TNFα-driven inflammation alone [19,20]. In this context, our findings support the concept that high pre-treatment 14-3-3 eta protein levels identify patients with a lower probability of early response to TNFα inhibition, reinforcing its potential utility as a biomarker for guiding therapeutic decision-making [21,22].

Baseline COMP levels were significantly lower in patients achieving a good clinical response to anti-TNFα therapy at 6 months, and COMP remained an independent contributor in the multivariate predictive model. These findings suggest that COMP reflects the structural component of disease activity, complementing inflammatory and immunological biomarkers. Longitudinal analysis revealed a delayed decrease in COMP levels, becoming significant only at 12 months, indicating that cartilage turnover may respond more slowly to biologic therapy than clinical inflammation. Higher pre-treatment COMP levels may therefore identify patients with more advanced structural involvement, in whom early clinical response is less pronounced despite TNFα inhibition. Previous studies suggested that lower baseline COMP levels may be associated with improved response to anti-TNF therapy, although this evidence remains limited and requires further validation [23].

An important observation of this study is the difference between predictors of treatment response at 6 months compared with 12 months. Several biomarkers demonstrated a clear predictive value at the early evaluation, whereas this association diminished at 12 months, when most patients achieved a good clinical response. This finding suggests that baseline immunological and structural biomarkers may be more informative for predicting early response to anti-TNFα therapy, while sustained treatment over time may attenuate baseline-related differences. Clinically, this highlights the relevance of early biomarker-based stratification when therapeutic decisions are most critical. Similarly, multi-biomarker composite scores such as the Vectra DA (MBDA) have been investigated as tools for predicting treatment response in rheumatoid arthritis. Some studies suggest that baseline MBDA scores or early changes in the score may be associated with subsequent clinical remission and treatment response, although overall results remain inconsistent, and their clinical utility is still under debate [24,25].

Beyond these biomarkers tested, several additional autoantibodies have been investigated as potential predictors of therapeutic response in RA. These include antibodies against carbamylated proteins (anti-CarP) and peptidylarginine deiminase (anti-PAD) enzymes. While some studies suggested associations between these autoantibodies and disease severity, radiographic progression, or differential response to biologic therapy, their predictive value remains inconsistent and has not yet been validated for routine clinical use. Collectively, these findings further support the notion that single autoantibody measurements are unlikely to fully capture the complexity of treatment response in RA, reinforcing the need for integrated, multi-biomarker approaches [26,27].

The challenge of identifying reliable biomarkers capable of predicting treatment response is not unique to RA but represents a broader issue across immune-mediated rheumatic diseases. In conditions such as psoriatic arthritis and spondyloarthritis, multiple studies have explored the predictive value of clinical, genetic, and serological markers for response to biologic therapies, yet results remain heterogeneous and often inconclusive. Similarly, in systemic lupus erythematosus, despite extensive investigation of autoantibody profiles and cytokine signatures, robust predictors of therapeutic response are still lacking [28,29,30].

The challenge for predicting response to biologic therapy extends beyond rheumatology and has been widely explored in other immune-mediated diseases, including inflammatory bowel disease and psoriasis. In inflammatory bowel disease, biomarkers such as C-reactive protein, fecal calprotectin, and therapeutic drug levels have been investigated as predictors of response to anti-TNF agents, although their predictive accuracy remains variable and context-dependent. Similarly, in psoriasis, several clinical, genetic, and immunological factors—including baseline disease severity, HLA-C*06:02 status, and cytokine profiles—have been associated with differential response to biologic therapies targeting TNFα, IL-17, or IL-23 pathways. Despite these advances, no single biomarker has demonstrated sufficient predictive performance for routine clinical use across these conditions. These findings further support the concept that treatment response in immune-mediated diseases is driven by complex, multifactorial mechanisms and reinforce the need for integrated, multi-biomarker approaches to improve patient stratification and therapeutic outcomes [31,32,33].

Real-world data further highlight the variability in long-term treatment persistence among patients with RA. Registry-based analyses have shown that retention rates of biologic and targeted synthetic DMARDs decrease over time, with a substantial proportion of patients requiring treatment modification due to insufficient response or loss of efficacy. These findings further emphasize the need for reliable biomarkers capable of supporting earlier and more individualized therapeutic decisions [34].

In addition to their potential role in predicting treatment response, biomarker-driven strategies may also contribute to optimizing long-term disease management by reducing unnecessary treatment switching and healthcare costs. Early identification of patients unlikely to respond to a given biologic agent could facilitate more timely therapeutic adjustments, potentially preventing prolonged exposure to ineffective therapies and limiting cumulative joint damage. Moreover, integrating biomarker profiles with clinical and imaging data may further refine risk stratification models and support a more dynamic, treat-to-target approach. As precision medicine continues to evolve in rheumatology, such integrative strategies may ultimately lead to improved patient outcomes and more efficient use of healthcare resources.

In line with our findings demonstrating a reduction in circulating autoantibody levels under biologic therapy, particularly among patients achieving a favorable clinical response, the concept of immunological remission has gained increasing attention in RA. Beyond clinical improvement, effective treatment may induce measurable changes in the underlying immune response, reflected by decreases in RF and, to a lesser extent, anti-CCP antibodies. Although complete normalization of autoantibody profiles is uncommon, declining titers have been associated with improved disease control and may reflect a partial restoration of immune tolerance. These observations suggest that dynamic monitoring of serological biomarkers could provide additional insight into treatment response and long-term disease control, supporting the potential role of immunological remission as a complementary therapeutic target in RA [35].

This study has several limitations, including the relatively small sample size, the single-center design, and the exclusive evaluation of TNFα inhibitors, which may limit the generalizability of the findings. In addition, due to the limited number of non-responders, the regression model may be subject to overfitting and should be interpreted with caution. The marked imbalance between responders and non-responders may also have influenced the apparent predictive performance and classification accuracy of the proposed model. These findings should therefore be considered exploratory and require validation in larger, independent cohorts. Future studies incorporating larger cohorts and external validation are needed to confirm the robustness of the proposed biomarker model. Additionally, although all evaluated biologic agents belonged to the anti-TNFα class, structural and pharmacodynamic differences between individual TNF inhibitors may have influenced treatment response and biomarker titers.

Nevertheless, the prospective design and standardized longitudinal assessment strengthen the clinical relevance of the results. From a practical perspective, the identification of baseline biomarkers associated with early treatment response may support more individualized therapeutic decision-making in patients with active RA.

In this context, the development of multiparametric biomarker panels integrating immunological, inflammatory, and clinical markers, may represent a key step toward a precision medicine approach in RA.

In our cohort, smokers were more frequently represented among non-responders at 6 months. Similar observations have been reported in previous studies evaluating treatment response to biologic therapy in RA.

## 5. Conclusions

Lower baseline serum levels of RF IgM and IgA, anti-CCP antibodies, and COMP were associated with a higher likelihood of achieving a good clinical response at 6 months in patients treated with anti-TNFα agents. Lower pre-treatment levels of 14-3-3 eta protein are also associated with good treatment response at 6 months.

Regarding treatment response at 12 months, reduced baseline serum levels of RF IgM and IgA demonstrated a predictive value for achieving a good clinical outcome.

The 12-month analysis should be interpreted with caution due to the very low number of non-responders, limiting statistical power.

Using univariate logistic regression analysis, baseline anti-CCP antibody levels and 14-3-3 eta protein levels were identified as significant predictors of treatment response at 6 months in patients receiving anti-TNFα therapy.

Furthermore, multivariate logistic regression analysis yielded a predictive model for treatment response at 6 months, incorporating baseline anti-CCP antibody levels, 14-3-3 eta protein levels, and COMP levels, with an overall classification accuracy of 89.1%.

Regarding the longitudinal evolution of serum biomarkers under biologic therapy, both RF isotypes (IgM and IgA) and anti-CCP antibody levels showed significant reductions at 6 and 12 months. In contrast, significant decreases in anti-MCV and COMP levels were observed only after 12 months of treatment. A progressive reduction in 14-3-3 eta protein levels was also noted at both follow-up visits; however, these changes did not reach statistical significance.

## Figures and Tables

**Table 1:** Baseline characteristics of study population.

Comorbidities	Sex	Place of Residence	BMI kg/m^2^ 27.06 ± 4.553	Smoking
HBP 37/64 (57.8%) Dyslipidemia 21/64 (32.8%) Diabetes 13/64 (20.3%)	Female 59/64 (92.2%)	Urban 27/64 (42.2%)	Height, cm 163.20 ± 7.391	Yes 12/64 (18.8%)
Osteoporosis 8/64 (12.5%)	Male 5/64 (7.9%)	Rural 37/64 (57.8%)	Weight, kg 72.18 ± 12.079	Former 4/64 (6.3%)
Osteoarthritis 6/64 (9.4%)				No 48/64 (75.0%)

**Table 2:** csDAMRDs therapies used before starting treatment with TNF alpha inhibitors.

First-Line csDMARDs		Second-Line csDMARDs		Gc	
HCQ	1/64 (1.6%)	LEF	25/64 (39%)	Methylprednisolone	11/21 (52.3%)
LEF	25/64 (39.1%)	HCQ	4/64 (6.25%)	Prednisone	10/21 (47.7%)
MTX	35/64 (54.7%)	SSZ	35/64(54.6%)		
SSZ	3/64 (4.7%)				

**Table 3:** Smoking status and response to treatment EULAR/2 groups at 6 months.

Smoking	6 Months Response Non-Responders	6 Months Response Moderate	6 Months Response Good	6 Months Response No	6 Months Response Yes
No	2/7 (28.6%)	29/38(76.3%)	17/19(89.5%)	2/7 (28.6%)	46/57 (80.7%)
Past	0/7 (0.0%)	3/38 (7.9%)	1/19 (5.3%)	0/7 (0.0%)	4/57 (7.0%)
Yes	5/7 (71.4%)	6/38 (15.8%)	1/19 (5.3%)	5/7 (71.4%)	7/57 (12.3%)
	*p* = 0.056			*p* = 0.017	

## Data Availability

The data presented in this study are available from the corresponding author upon reasonable request. The data are not publicly available due to ethical and privacy restrictions.

## Abbreviations

The following abbreviations are used in this manuscript:

ACPA	Anti-citrullinated protein antibodies
anti-CCP	Anti-cyclic citrullinated peptide antibodies
anti-MCV	Anti-mutated citrullinated vimentin antibodies
bDMARDs	Biologic disease-modifying antirheumatic drugs
COMP	Cartilage oligomeric matrix protein
CRP	C-reactive protein
csDMARDs	Conventional synthetic disease-modifying antirheumatic drugs
DAS28-CRP	Disease Activity Score in 28 joints based on C-reactive protein
ELISA	Enzyme-linked immunosorbent assay
ESR	Erythrocyte sedimentation rate
EULAR	European Alliance of Associations for Rheumatology
IgA	Immunoglobulin A
IgM	Immunoglobulin M
MTX	Methotrexate
LEF	Leflunomide
SSZ	Sulfasalazine
HCQ	Hydroxychloroquine
Gc	Glucocorticoids
HBP	High blood pressure
RA	Rheumatoid arthritis
RF	Rheumatoid factor
TNFα	Tumor necrosis factor alpha
